# Investigations on the Wound Healing Potential of Tilapia Piscidin (TP)2-5 and TP2-6

**DOI:** 10.3390/md20030205

**Published:** 2022-03-10

**Authors:** Chia-Wen Liu, Chu-Yi Hsieh, Jyh-Yih Chen

**Affiliations:** Marine Research Station, Institute of Cellular and Organismic Biology, Academia Sinica, 23-10 Dahuen Road, Jiaushi, Ilan 262, Taiwan; culex763@gmail.com (C.-W.L.); joy.cde49@gmail.com (C.-Y.H.)

**Keywords:** wound healing, antimicrobial peptide, tilapia piscidin

## Abstract

Wound healing is a highly orchestrated process involving many cell types, such as keratinocytes, fibroblasts and endothelial cells. This study aimed to evaluate the potential application of synthetic peptides derived from tilapia piscidin (TP)2, TP2-5 and TP2-6 in skin wound healing. The treatment of HaCaT keratinocytes with TP2-5 and TP2-6 did not cause cytotoxicity, but did enhance cell proliferation and migration, which could be attributed to the activation of epidermal growth factor receptor signaling. In CCD-966SK fibroblasts, although TP2-5 (31.25 μg/mL) and TP2-6 (125 μg/mL) showed cytotoxic effects, we observed the significant promotion of cell proliferation and migration at low concentrations. In addition, collagen I, collagen III, and keratinocyte growth factor were upregulated by the peptides. We further found that TP2-5 and TP2-6 showed pro-angiogenic properties, including the enhancement of human umbilical vein endothelial cell (HUVEC) migration and the promotion of neovascularization. In a murine model, wounds treated topically with TP2-5 and TP2-6 were reduced by day 2 post-injury and healed significantly faster than untreated wounds. Taken together, these findings demonstrate that both TP2-5 and TP2-6 have multifaceted effects when used as topical agents for accelerating wound healing.

## 1. Introduction

The skin provides a strong protective barrier against the environment, and any disruption of skin integrity must be rapidly and efficiently repaired. The healing of skin wounds is a specialized, complicated physiological process that involves a variety of cells and factors [1,2]. Classical wound healing involves an initial inflammatory reaction, followed by a period of skin regeneration that includes re-epithelialization, granulation tissue formation, and angiogenesis [3]. During the re-epithelialization process, keratinocytes proliferate and migrate to the wound in order to restore the integrity of the epidermis and form a barrier against microbial pathogens [4,5]. Meanwhile, dermal fibroblasts migrate and proliferate to close the wound, producing extracellular matrix (ECM) components, such as collagen I and III, that form new granulation tissue [6]. During this process, fibroblasts also secrete growth factors, such as keratinocyte growth factor (KGF), that crosstalk with keratinocytes and other effector cells to facilitate re-epithelialization and wound contraction [7]. While new tissue is being generated, proangiogenic factors such as basic fibroblast growth factor (bFGF) stimulate endothelial cells to migrate into the region and form new vasculature in the process of angiogenesis [8]. The prolongation or deregulation of any of these processes during wound healing can lead to the formation of chronic non-healing wounds [9,10].

Antimicrobial peptides (AMPs) are multifunctional molecules that have rapid and broad-spectrum antimicrobial activities against bacteria, fungi and some viruses, and they are also known to modulate the host immune response [11,12]. Some endogenous and synthetic AMPs not only display direct antimicrobial effects, but also show effects on the cells involved in wound healing [13]. For instance, human cathelicidin (LL-37) can protect human keratinocytes from apoptosis and induce cell proliferation [14,15]. Additionally, Temporins A and B, which are frog skin AMPs, promote the proliferation and migration of endothelial cells [16]. Moreover, the enhanced synthesis of the extracellular matrix promotes faster wound healing after treatment with CaTx-II, an AMP from snake venom [17]. Previously, five piscidin-like peptides were identified from *Oreochromis niloticus* and named tilapia piscidin (TP)1-5 [18]. Among these AMPs, TP3 and TP4 exhibited especially potent antimicrobial activities. However, their relatively high cytotoxicities and hemolytic activities have severely restricted the clinical application of the molecules [18,19]. To overcome such issues, we recently developed two peptides derived from the sequence of TP2, called TP2-5 and 2-6. The redesigned peptides exhibit effective antimicrobial and anti-biofilm activities with improved hemolytic activity and cytotoxic profiles [20]. These features suggest that TP2-5 and TP2-6 could be suitable for development as antimicrobial agents. However, the therapeutic potential of TP2-5 and TP2-6 in regenerative medicine remains unclear. Thus, this study aimed to evaluate the utility of TP2-5 and TP2-6 in promoting wound healing processes in vitro and accelerating wound closure in vivo.

## 2. Results

### 2.1. Tilapia Piscidin (TP)2-5 and TP2-6 Induce Cell Proliferation and Motility of Skin Keratinocytes

**Figure 1:**
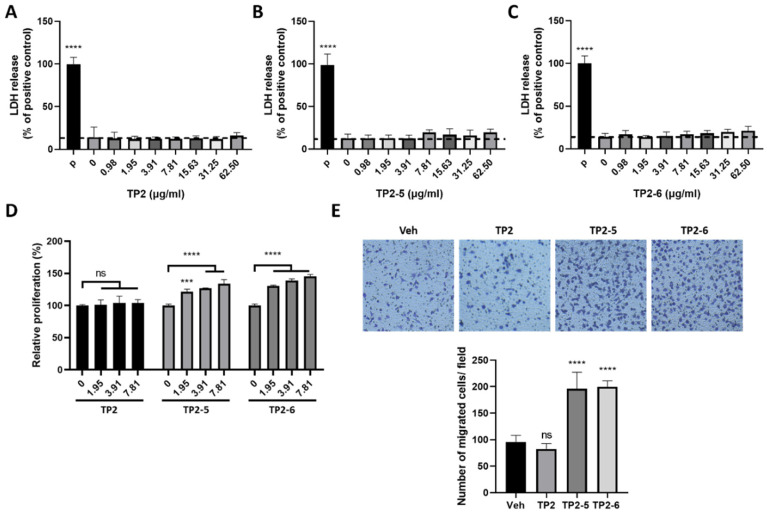
Treatment with tilapia piscidin (TP)2-5 or TP2-6 promotes the proliferation and migration of HaCaT cells. (**A**–**C**) HaCaT cells were treated with different doses (0.98, 1.95, 3.91, 7.81, 15.63, 31.25, 62.5 μg/mL) of TP2 (**A**), TP2-5 (**B**) or TP2-6 (**C**) for 24 h, and the LDH release assay was performed. The positive control was 0.1% Triton-X 100 (P). Results are shown as percentages of the positive control. (**D**) The proliferation of HaCaT cells was assessed by MTS/PMS assays. HaCaT cells were separately incubated with different doses (0, 1.95, 3.91, or 7.81 μg/mL) of TP2, TP2-5, or TP2-6 for 72 h. Statistical comparisons were made between various concentrations of peptides and 0 μg/mL peptides. (**E**) HaCaT cells were pretreated with 5 μg/mL mitomycin C for 2 h and afterward stimulated with 3.91 μg/mL of TP2, TP2-5, or TP2-6, followed by a transwell migration assay. The cells that migrated towards the lower side of the membrane were fixed with 4% paraformaldehyde and stained with 0.1% crystal violet, then counted in 5 independent fields per condition. Representative images are shown. Data are presented as mean ± SD. *** *p* < 0.001, **** *p* < 0.0001, ns, not statistically significant versus the vehicle control.

According to the lactate dehydrogenase (LDH) release assay, neither TP2-5 nor TP2-6 showed cytotoxic effects on human skin keratinocytes (HaCaT cells) for peptide concentrations up to 62.5 μg/mL, similar to the parental peptide, TP2 (Figure 1A–C). Next, we tested the capacity of the peptides to promote keratinocyte proliferation and migration. HaCaT cells were treated with several concentrations (1.95, 3.91, and 7.81 μg/mL) of peptide for 72 h, and cell proliferation was dose-dependently increased by both TP2-5 and TP2-6 (Figure 1D). Notably, TP2 did not affect the growth of HaCaT cells (Figure 1D). Furthermore, the keratinocyte’s migratory ability under TP2-5 or TP2-6 treatment was investigated using a transwell migration assay. Cells were pretreated with mitomycin C to exclude interference by the proliferative effects of the peptides [21]. The cultured HaCaT cells and 3.91 μg/mL of TP2, TP2-5, or TP2-6 were added to the upper transwell chambers. After incubation, we found that treatment with TP2-5 and TP2-6 caused an approximate 2-fold increase in keratinocyte migration compared with vehicle control and TP2 treatment (Figure 1E). These results indicate that the treatment of TP2-5 or TP2-6 not only induces cell proliferation, but also improves the migratory ability of keratinocytes.

### 2.2. Epidermal Growth Factor Receptor (EGFR) Signaling Is Involved in TP2-5- and TP2-6-Enhanced Keratinocyte Proliferation and Motility

**Figure 2:**
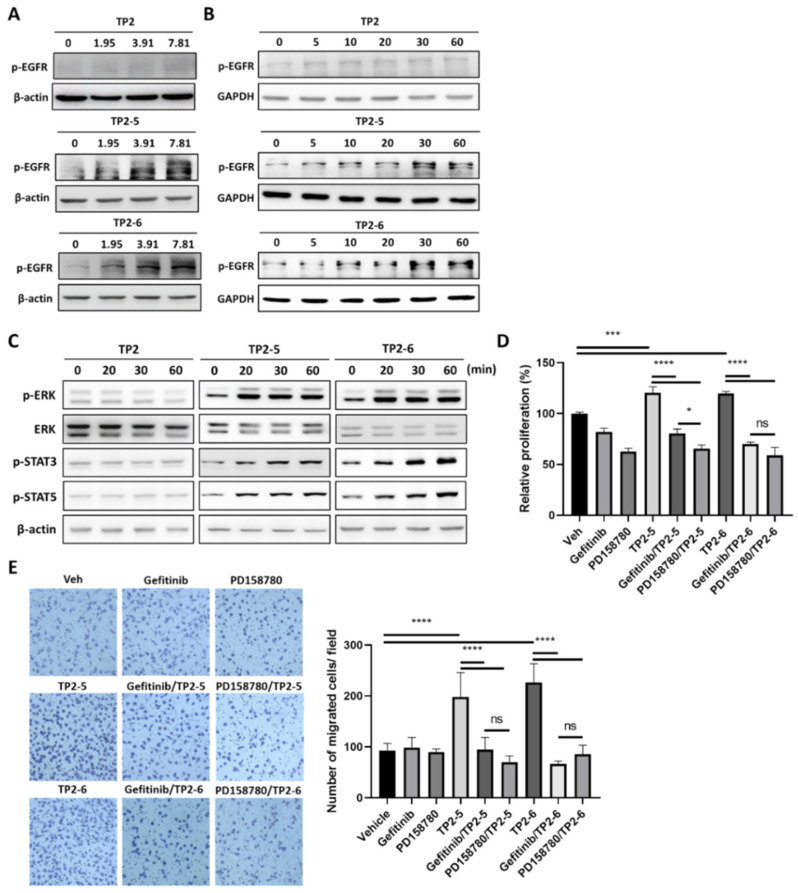
TP2-5 and TP2-6 induce epidermal growth factor receptor (EGFR) activation, which contributes to keratinocyte proliferation and migration. (**A**) Sub-confluent HaCaT cells were starved for 12 h and afterward treated with 1.95, 3.91, or 7.81 μg/mL of TP2 (upper blots), TP2-5 (middle), or TP2-6 (lower) for 30 min, and then EGFR phosphorylation (p-EGFR) was detected by immunoblotting. β-actin was used as the loading control. (**B**) Serum-starved HaCaT cells were stimulated with 3.91 μg/mL of TP2 (upper blots), TP2-5 (middle), or TP2-6 (lower), and harvested into lysis buffer at the indicated times (min). Phosphorylated EGFR (p-EGFR) levels were detected by immunoblotting. GAPDH was used as the loading control. (**C**) The detection of phosphorylated ERK (p-ERK), STAT3 (p-STAT3), and STAT5 (p-STAT5) after 3.91 μg/mL of TP2 (left), TP2-5 (middle), or TP2-6 (right) treatment in HaCaT cells at the indicated times (min) by immunoblotting. ERK and β-actin were used as the loading control. (**D**) Serum-starved HaCaT cells were pretreated with the tyrosine kinase inhibitors, gefitinib (10 µM) or PD158780 (10 µM) for 2 h and then incubated with 3.91 μg/mL of TP2-5 or TP2-6 for 72 h. The proliferation of HaCaT cells was assessed by the MTS/PMS assay. (**E**) Serum-starved HaCaT cells were pretreated with 5 μg/mL mitomycin C and with or without tyrosine kinase inhibitor, gefitinib (10 µM) or PD158780 (10 µM) for 2 h. Then the transwell migration assay was performed under 3.91 μg/mL of TP2-5 or TP2-6. The cells that migrated towards the lower side of the membrane were fixed with 4% paraformaldehyde, stained with 0.1% crystal violet, and then counted within five independent fields per condition. Representative images are shown. Data are presented as mean ± SD. * *p* < 0.05, *** *p* < 0.001, **** *p* < 0.0001, ns, not statistically significant versus the vehicle control.

EGFR and its downstream signaling activation contribute to the proliferation and migration of keratinocytes [22,23]. Thus, we wanted to investigate whether EGFR signaling was stimulated by the TP2-5 or TP2-6 treatment of HaCaT cells. As shown in Figure 2A,B, HaCaT cells were stimulated with TP2, TP2-5 and TP2-6 using different concentrations (1.95, 3.91, 7.81 μg/mL) and for different amounts of time (5, 10, 20, 30, 60 min). Surprisingly, we found that the treatment of TP2-5 and TP2-6 resulted in dose- and time-dependent increases in EGFR phosphorylation (p-EGFR). In contrast, the effect of TP2 on p-EGFR level was not significant. Moreover, we observed that TP2-5 and TP2-6, but not TP2, strongly activated EGFR downstream signaling, including extracellular signal-regulated kinase (ERK), signal transducer and activator of transcription (STAT) 3, and STAT5 (Figure 2C). Next, we sought to gain further insight into the role of EGFR signaling activation in TP2-5- and TP2-6-promoted HaCaT cell proliferation and migration. Cells were pretreated with small-molecule tyrosine kinase inhibitors, PD158780 (pan-ErbB inhibitor; 10 μM) or gefitinib (EGFR inhibitor; 10 μM), to suppress EGFR activation (Supplementary Figure S1A,B). Peptide-promoted cell proliferation (Figure 2D) and migration (Figure 2E) were completely abolished in the presence of inhibitors, demonstrating that TP2-5 and TP2-6 induce keratinocyte proliferation and migration via EGFR activation.

### 2.3. TP2-5 and TP2-6 Stimulate Dermal Fibroblast Proliferation, Migration, and Collagen Synthesis

**Figure 3:**
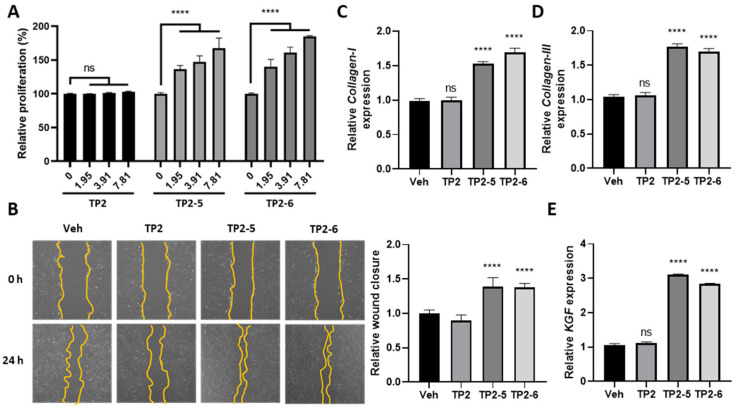
TP2-5 and TP2-6 stimulate the proliferation, migration, and extracellular matrix synthesis of CCD-966SK cells. (**A**) CCD-966SK cells were incubated with different doses (1.95, 3.91, or 7.81 μg/mL) of TP2, TP2-5, or TP2-6 for 72 h. The proliferation of cells was assessed by MTS/PMS assays. Statistical analysis was performed for various concentrations of peptides down to 0 μg/mL peptides. (**B**) CCD-966SK cells were pretreated with 5 μg/mL mitomycin C and afterward scratched and stimulated with PBS vehicle (Veh), and 3.91 μg/mL of TP2, TP2-5, or TP2-6. The extent of wound closure was calculated by analyzing the scratched area recovered by the cells after 24 h using ImageJ software. The data were normalized to the vehicle control values. (**C**,**D**) Relative expression levels of Collagen-I (**C**) and Collagen-III (**D**) in CCD-966SK cells under PBS vehicle (Veh), or 3.91 μg/mL of TP2, TP2-5, or TP2-6 treatment, were measured by qRT-PCR. (**E**) Relative expression of keratinocyte growth factor (KGF) in CCD-966SK cells under PBS vehicle (Veh), or 3.91 μg/mL of TP2, TP2-5, or TP2-6 treatments, were measured by qRT-PCR. Data are presented as mean ± SD. **** *p* < 0.0001, ns, not statistically significant versus the vehicle control.

The activation of dermal fibroblasts plays an important role in the skin reparative process [24]. TP2-5- and TP2-6-treated skin fibroblasts (CCD-966SK cells) caused significant LDH release at 31.25 and 125 μg/mL, respectively [20]. However, low concentrations of TP2-5 and TP2-6, ranging from 1.95 to 7.81 μg/mL, significantly promoted cell proliferation after 72 h incubation (Figure 3A). To further test the ability of TP2-5 or TP2-6 to provoke fibroblast migration, a scratch healing assay was performed in the presence of mitomycin C. Both TP2-5 and TP2-6 (3.91 μg/mL) significantly promoted the migration of CCD-966SK cells (Figure 3B). In contrast, neither cell proliferation nor migration was affected by TP2. The synthesis of ECM components is required for the recovery of skin flexibility and cell adhesion [6]. We found that TP2-5 or TP2-6 treatment upregulates the mRNA expression levels of ECM components, collagen I and III (Figure 3C,D). Furthermore, keratinocyte growth factor (KGF) is a crucial mediator of crosstalk between fibroblasts and keratinocytes [25]. KGF was also upregulated by TP2-5 and TP2-6 treatment (Figure 3E). Notably, treatment with TP2 did not affect the expressions of these genes.

### 2.4. TP2-5 and TP2-6 Promote Vascular Endothelial Cell Migration and Angiogenesis in Chicken Chorioallantoic Membrane (CAM) Assay

**Figure 4:**
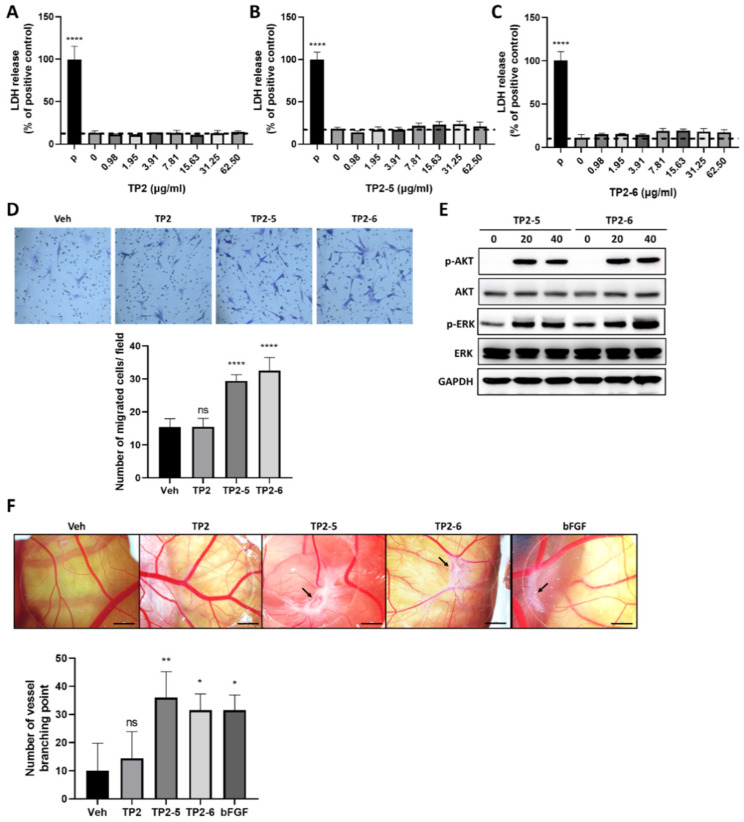
TP2-5 and TP2-6 promote human umbilical vein endothelial cells HUVEC migration and angiogenesis in the chicken chorioallantoic membrane (CAM) assay. (**A**–**C**) HUVECs were treated with different doses (0.98, 1.95, 3.91, 7.81, 15.63, 31.25, 62.5 μg/mL) of TP2 (**A**), TP2-5 (**B**) or TP2-6 (**C**) for 24 h and subjected to the LDH release assay. The positive control was 0.1% Triton-X 100 (P). Results are shown as relative percentages of the positive control. (**D**) HUVECs were pretreated with 5 μg/mL mitomycin C for 2 h, followed by the transwell migration assay under PBS vehicle (Veh), or 3.91 μg/mL of TP2, TP2-5, or TP2-6 treatment. The cells that migrated toward the lower side of the membrane were fixed with 4% paraformaldehyde and stained with 0.1% crystal violet, and then counted in five independent fields per condition. Representative images and quantitative results are shown. (**E**) Sub-confluent HUVECs were starved for 12 h, and afterward treated with 3.91 μg/mL of TP2-5 or TP2-6. The cells were harvested into lysis buffer at the indicated times (min) and we detected the total and phosphorylated AKT (AKT/p-AKT) and ERK (ERK/p-ERK) by immunoblotting. GAPDH was used as the loading control. (**F**) A CAM assay was used to determine the effects of TP2, TP2-5, and TP2-6 on angiogenesis ex vivo. Representative images show the appearance of blood vessels in chick embryo CAMs treated with vehicle (Veh) or TP2, TP2-5, or TP2-6 (5 μg/egg). bFGF (50 ng/egg) served as the positive control. The arrow indicates the formation of a wheel–spoke vessel structure; bar is 2 mm. The number of vessel branching points was counted and is expressed as the mean ± SD. * *p* < 0.05, ** *p* < 0.01, **** *p* < 0.0001, ns, not statistically significant versus the vehicle control.

Vascular endothelial cells are the principal cells of blood vessels, and their migratory ability is essential for the formation of new blood vessels during angiogenesis [8]. As shown in Figure 4A–C, TP2, TP2-5 and TP2-6 had no cytotoxic effects on human umbilical vein endothelial cells (HUVECs). Using the transwell migration assay with preincubation of mitomycin C, the cultured HUVECs were treated with vehicle or 3.91 μg/mL of TP2, TP2-5 or TP2-6 and added to the upper transwell chambers. We found a significantly enhanced migratory activity of HUVECs upon treatment with either TP2-5 or TP2-6 (3.91 μg/mL) (Figure 4D). Furthermore, it is well known that the cellular activation of both phosphatidylinositol 3-kinase (PI3K)/Akt and mitogen/extracellular signal-regulated kinase (MEK)/ERK signaling pathways contribute to the activation of angiogenesis [26]. Indeed, the immunoblotting analysis of TP2-5- (3.91 μg/mL) or TP2-6 (3.91 μg/mL)-treated HUVECs indicated notable increases in phosphorylated AKT (p-AKT) and ERK (p-ERK) levels (Figure 4E), suggesting that TP2-5 and TP2-6 stimulate angiogenesis. To further confirm the angiogenic effects of TP2-5 and TP2-6, the peptides (5 μg/egg) were tested in the CAM assay. As shown in Figure 4F, compared with the vehicle control and TP2, both TP2-5 and TP2-6 induced more vessel branching points after 3 days of treatment, as evidenced by the wheel–spoke vessel formation that was similar to the positive control, bFGF (50 ng/egg) [27].

### 2.5. Effects of TP2-5 and TP2-6 on Wound Healing in Mice

**Figure 5:**
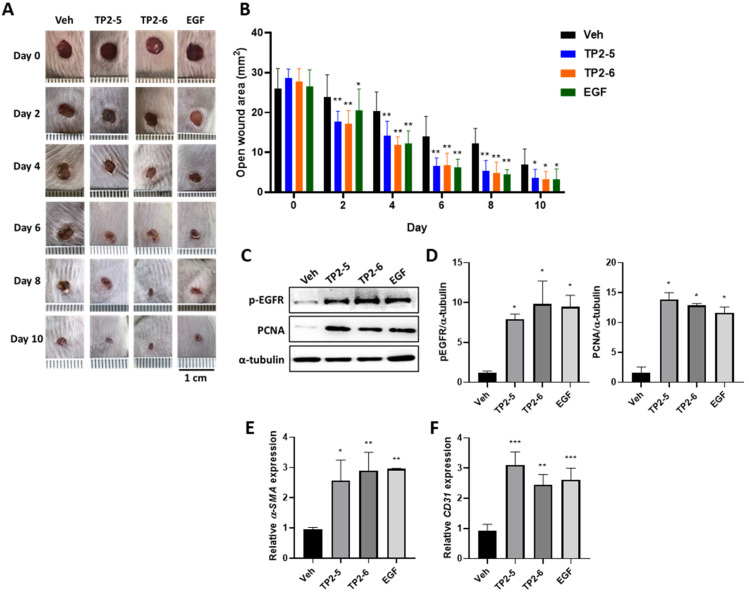
Topical application of TP2-5 and TP2-6 accelerates the healing of full-thickness wounds in mice. BALB/c mice were dorsally wounded and topically treated with PBS vehicle (Veh), TP2-5, TP2-6, or EGF (2 μg/wound; *n* = 6) four times every day. (**A**) Representative photographs show the macroscopic wounds on different days post-injury. Scale bar = 1 cm. (**B**) Measurements of the open wound area (mm^2^) were made using ImageJ. (**C**) Protein levels of p-EGFR and proliferating cell nuclear antigen (PCNA) in the full-thickness wound tissues were detected by immunoblotting at day 4 post-injury. (**D**) Band intensities were quantified with ImageJ. Levels of p-EGFR and PCNA were normalized to α-tubulin (*n* = 3). (**E**,**F**) Relative expression levels of α-smooth muscle actin (α-SMA) (**E**) and platelet endothelial cell adhesion molecule-1 (CD31) (**F**) in the full-thickness wound tissues were measured by qRT-PCR at day 4 post-injury (*n* = 3). Data are presented as mean ± SD * *p* < 0.05, ** *p* < 0.01, *** *p* < 0.001 versus the vehicle control group.

Considering that both TP2-5 and TP2-6 showed the multifaceted promotion of wound healing in HaCaT, CCD-966SK, and HUVEC cultures, we next evaluated whether the topical application of TP2-5 and TP2-6 would modify the healing of full-thickness skin wounds in a mouse model. Mice were dorsally wounded and topically treated with PBS vehicle (Veh), TP2-5, TP2-6, or EGF (2 μg/wound; *n* = 8) four times every day. As shown in Figure 5A,B, wound healing in TP2-5- and TP2-6-treated mice was accelerated by day 2 post-injury. On day 4, the TP2-5 and TP2-6 groups had open wound areas that were decreased by more than 50%, similar to the EGF-treated positive control group. On day 10, the wounds of TP2-5-, TP2-6- and EGF-treated mice were almost completely closed, whereas the wounds of control mice were not. No adverse effects on the general health or behavior of the mice were observed for any treatment group. To further confirm the observations made in culture, the protein levels of p-EGFR and the proliferating cell nuclear antigen (PCNA; a cell proliferation marker) were evaluated by immunoblotting at day 4 post-injury (Figure 5C,D). Consistently, both TP2-5 and TP2-6 could promote p-EGFR and PCNA expression in full-thickness wound tissues. We also observed that the activated fibroblast marker, α-smooth muscle actin (α-SMA) (Figure 5E), and the angiogenesis marker, platelet endothelial cell adhesion molecule-1 (CD31) (Figure 5F), were induced in TP2-5- and TP2-6-treated wound tissues. These results demonstrate that topically applied TP2-5 and TP2-6 have high potential applicability as skin wound treatments.

## 3. Discussion

Wound healing is a fundamental process in re-establishing tissue integrity [28]. Impairments in this healing process can aggravate the disease and have a significant impact on quality of life [29]. Antimicrobial peptides (AMPs) are natural antibiotics recognized for their potent antibacterial and wound healing properties [13,30]. Recently, we developed two novel piscidin-like peptides, TP2-5 and TP2-6, which possess antibacterial and anti-biofilm activities against broad-spectrum bacteria [20]. In this study, we demonstrate that TP2-5 and TP2-6 promote wound healing processes in various types of skin cells, including keratinocytes, fibroblasts, and endothelial cells, along with low cytotoxic effects. In addition, the treatments with TP2-5 and TP2-6 accelerated the closure of full-thickness skin wounds in mice.

According to our data, TP2-5 and TP2-6 elicited positive effects on keratinocyte proliferation, while TP2 did not (Figure 1D). In addition, keratinocyte’s migratory ability was also stimulated by TP2-5 and TP2-6 (Figure 1E). Rapid keratinocyte proliferation and migration promote the efficiency of the initial wound healing process [31,32]. The activation of epidermal growth factor receptor (EGFR) and downstream molecules are known to directly trigger these actions [33]. We found that treatment with TP2-5 and TP2-6 rapidly increase EGFR phosphorylation, and blocking EGFR activation almost completely abrogated the peptide-promoted migration and proliferation of keratinocytes (Figure 2). Upon EGFR activation, ERK and STAT3 act as key mediators of proliferation and migration in various cell types [34,35,36]. Our results are similar to previous reports on the effects of human β-defensins and LL-37, which are epithelial cell-derived AMPs that also increase peptide-mediated ERK and STAT3 phosphorylation in keratinocytes [15,37]. Furthermore, STAT5 is specifically activated by EGFR transactivation via the metalloprotease-mediated shedding of membrane-anchored EGFR ligands, such as heparin-binding (HB)-EGF [38,39]. The downregulation of STAT5 by siRNA reduces keratinocyte proliferation and migration promoted by HB-EGF [38]. We found that apart from the effects on ERK and STAT3, TP2-5 and TP2-6 also clearly elevated the level of phosphorylated STAT5 (Figure 2C), implying that EGFR transactivation may be triggered by TP2-5 and TP2-6 treatment. LL-37 has been found to cleave HB-EGF through the activation of metalloproteinase, which phosphorylates EGFR and induces keratinocyte proliferation and migration [15,40]. Melittin [41] and Pep19-2.5 [42] were found to induce metalloprotease-mediated EGFR transactivation through the activation of the G-protein-coupled receptor, P2 × 7, which leads to keratinocyte migration. However, the mechanism by which TP2-5 and TP2-6 activate EGFR phosphorylation during skin wound healing requires further investigation.

Fibroblasts support normal wound healing by infiltrating the wounded tissue within 24–48 h and dissolving the fibrin–fibronectin clot before replacing it with a collagen matrix [43,44]. It has been found that collagen deposition is impaired in diabetic wounds due to decreased fibroblast proliferation and migration [45,46]. Here, we observed that TP2-5 and TP2-6 not only promote cell proliferation and migration, but they also stimulate the expression of collagen I and III in fibroblasts [20] (Figure 3). Furthermore, previous studies have reported a double paracrine network in which the KGF secreted from fibroblasts promotes keratinocyte proliferation and migration, and stimulates the keratinocyte secretion of TGF-β1 [25]. The secreted TGF-β1 in turn promotes fibroblast differentiation into myofibroblasts [47,48]. We found that KGF mRNA expression is upregulated by TP2-5 and TP2-6 treatment in fibroblasts, which implies that in addition to their direct effects on fibroblasts, TP2-5 and TP2-6 may assist the growth factor-mediated crosstalk between keratinocytes and fibroblasts.

The formation of new blood vessels is essential for tissue repair, because the vessels can support cells at the wound site by supplying nutrition and oxygen [49]. The angiogenic process involves a cascade of events, with the migration of endothelial cells serving as an important component [50]. Here, we found that TP2-5 and TP2-6 showed pro-angiogenic properties by promoting HUVEC migration (Figure 4D). The results were further supported by an ex vivo CAM assay, which showed more vessel branching points and the formation of wheel–spoke vessels in the TP2-5- and TP2-6-treated groups versus the control group (Figure 4F). MEK/ERK and PI3K/AKT signals have been extensively studied regarding endothelial cell migration. The activation of the MEK/ERK pathway in endothelial cells regulates the abundance of proteins such as paxillin and focal adhesion kinase, which are required for cell migration [51,52,53,54], while the activation of the AKT pathway can increase vascular endothelial growth factor secretion and modulate the expression of other angiogenic factors, such as nitric oxide and angiopoietins [55,56]. Our data demonstrate that TP2-5 and TP2-6 promote ERK and AKT phosphorylation in endothelial cells (Figure 4E), strongly implying that the activation of the ERK and AKT signaling pathways might be responsible for TP2-5- and TP2-6-mediated endothelial cell migration and new blood vessel formation.

Our previous report showed that TP2-5 and TP2-6 exert effective antimicrobial and antibiofilm activities against *Acinetobacter baumannii* (*A. baumannii*), even multidrug-resistant strains [20]. *A. baumannii* is a major cause of wound infections in patients who are critically ill, and its presence delays wound healing and promotes the development of sepsis [57,58]. In this study, we found that TP2-5 and TP2-6 markedly enhanced the migration and proliferation of skin cells in vitro with low cytotoxicity (Figure 1, Figure 3 and Figure 4). Furthermore, the topical application of TP2-5 and TP2-6 greatly accelerated full-thickness skin wound closure in a mouse model (Figure 5). These results demonstrate that TP2-5 and TP2-6 may have potential utility in the treatment of infected wounds. Further studies are planned to further investigate this potential therapeutic benefit.

## 4. Materials and Methods

### 4.1. Cell Culture

Human skin keratinocytes (HaCaT, CVCL_0038), skin fibroblasts (CCD-966SK, CVCL_U267), and human umbilical vein endothelial cells (HUVECs, CRL-1730) were obtained from the Bioresource Collection and Research Center (Hsinchu, Taiwan) and maintained in Dulbecco’s modified Eagle’s medium (DMEM; GIBCO Inc., Brooklyn, NY, USA) supplemented with 10% heated-inactivated fetal bovine serum (FBS), penicillin (100 U/mL), and streptomycin (100 μg/mL) (Hyclone, Logan, UT, USA). All cell lines were cultured in 5% CO_2_ at 37 °C.

### 4.2. Peptides

TP2 (GECIWDAIFHGAKHFLHRLVNP), TP2-5 (KKCIAKAILKKAKKLLKKLVNP), and TP2-6 (KKCIAKAILKKAKKLLKDLVNP) were synthesized by GL Biochem (Shanghai, China) and diluted in sterile PBS before use. The preparations were filtered through a syringe filter (PES membrane, pore size 0.22 μm; Molecular Devices, Sunnyvale, CA, USA) for bacterial sterilization.

### 4.3. Lactate Dehydrogenase (LDH) Release Assay

The cytotoxicity of the peptides was analyzed by LDH release assays. HaCaT and HUVEC cells were seeded on 96-well cell culture plates (Corning Inc., Corning, OH, USA) and treated with the indicated concentrations of TP2, TP2-5, or TP2-6 for 24 h. 0.1% Triton-X 100 (Sigma-Aldrich, St. Louis, MO, USA) served as the positive control. After treatment, the culture supernatants were collected in a Cytotoxicity Detection Kit (LDH) (Roche Diagnostics, Mannheim, Germany) according to the manufacturer’s protocol. Briefly, 100 μL of cell supernatants were incubated with 100 μL LDH reaction mix for 10 min and then incubated with 50 μL of stop solution for 15 min at room temperature. The optical density at 492 nm was measured on a SpectraMax i3 Multi-Mode Microplate Reader (Molecular Devices, Sunnyvale, CA, USA).

### 4.4. MTS/PMS Assay

The proliferation of HaCaT and CCD-966SK cells was analyzed with MTS/PMS assays. Cells were seeded on 96-well cell culture plates (Corning Inc., Corning, OH, USA) and treated with the indicated concentrations of TP2, TP2-5, or TP2-6 for 72 h. Then, 20 μL of MTS/PMS mixture (20:1) reagent (Promega, Madison, WI, USA) and 80 μL of cell growth medium were added to each well, followed by incubation at 37 °C for 1 h. The optical density at 490 nm was measured on a SpectraMax i3 Multi-Mode Microplate Reader. For treatment of the tyrosine kinase inhibitor, sub-confluent HaCaT cells were starved for 12 h in DMEM without 10% FBS and incubated with 10 µM gefitinib or 10 µM PD158780 (Sigma-Aldrich, St. Louis, MO, USA) for 2 h in DMEM without 10% FBS before peptide treatment.

### 4.5. Cell Migration Assay

HaCaT and HUVEC migration was assessed with a transwell migration assay, using a transwell chamber (8.0 μm pore size; Corning Inc., Corning, OH, USA). The cells were incubated with 5 μg/mL mitomycin C (Roche, Philadelphia, PA, USA) for 2 h prior to the assay to prevent cell proliferation. The lower compartment was filled with DMEM containing 10% FBS. The upper compartment was filled with cells (3 × 10^4^) resuspended in serum-free DMEM containing the indicated treatments. After incubation at 37 °C for 12 h, cells in the upper chamber were carefully removed with a cotton swab, and the migrating cells on the lower membrane surface were fixed with 4% paraformaldehyde (Sigma-Aldrich, St. Louis, MO, USA) for 15 min and stained with 0.1% crystal violet (Sigma-Aldrich, St. Louis, MO, USA) for 20 min. For quantification, five fields per chamber were counted under a microscope (Leica, Hamburg, Germany).

To detect and quantify dermal fibroblast migration, a scratch healing assay was used. CCD-966SK cells were seeded into six-well plates (Corning Inc., Corning, OH, USA) and pre-incubated with 5 μg/mL mitomycin C (Roche) for 2 h before the assay to prevent cell proliferation. A linear scratch was made on cell monolayers with a sterile pipette, and cultures were incubated in growth medium containing vehicle PBS (Veh), TP2, TP2-5, or TP2-6 (3.91 μg/mL). The extent of wound closure was calculated by analyzing the scratched area covered by the cells after 24 h using ImageJ software. The data were normalized to the vehicle control values.

### 4.6. Quantitative Real-Time PCR (qRT-PCR) Analysis

Total RNA was extracted from treated CCD-966SK cells or homogenized skin tissue isolated from mouse wound sites using TRIzol Reagent (Invitrogen, Carlsbad, CA, USA), and 1 μg total RNA from each sample was reverse-transcribed into cDNA with RT-PCR Quick Master Mix (Toyobo, Tokyo, Japan), according to the manufacturer’s instructions. A StepOne Plus Real-Time QPCR System (Applied Biosystems, Foster City, CA, USA) was used for qRT-PCR. The reaction mixture was prepared in a final volume of 10 μL per reaction. Briefly, 1 μL of sample cDNA was added to 9 μL of a master mix containing 5 μL of SYBR Green Real-Time PCR Master Mix (Toyobo, Osaka, Japan) and 4 μL of nuclease-free water (Sigma-Aldrich, St. Louis, MO, USA) with primers at a final concentration of 0.5 μM. The following primers (synthesized by Genomics BioSci and Tech, Taipei, Taiwan) were used: human Collagen I: 5′- GCCAAGACGAAGACATCCCA-3′ (forward), 5′-CCACACGTCTCGGTCATGG-3′ (reverse); human Collagen III: 5′-TGGTGTTGGAGCCGCTGCCA-3′ (forward), 5′-CTCAGCACTAGAATCTGTCC-3′ (reverse); human KGF: 5′-CTGCTCTATAATGCGCAAATGG-3′ (forward), 5′-GAGGTGGAAGCACGGTCTGT-3′ (reverse); human GAPDH: 5′-TCAGCAATGCCTCCTGCAC-3′ (forward), 5′-GTGATGGCATGGACTGTGGTC-3′ (reverse); mouse α-SMA: 5′-CTTCGCTGAGCAGATTGGCTGT-3′ (forward), 5′-ACTCCTCTTGCTTGGCCACCT-3′ (reverse); mouse CD31: 5′-CCAAAGCCAGTAGCATCATGGTC-3′ (forward), 5′-GGATGGTGAAGTTGGCTACAGG-3′ (reverse); mouse GAPDH: 5′- GAGCGAGACCCCACTAACAT-3′ (forward), 5′- TCTCCATGGTGGTGAAGACA-3′ (reverse). The thermal cycling program was carried out according to the manufacturer’s instructions. The data were collected in triplicate, and gene expression levels were calculated by normalizing the cycle threshold (Ct) values of the target gene to the Ct values of the internal reference gene, GAPDH, (ΔCt), determined as 2^−^^ΔCt^.

### 4.7. Immunoblotting

After performing specific treatments, cell cultures or skin tissue isolated from mouse wound sites were harvested and washed three times with PBS; total protein lysates were prepared using RIPA lysis buffer (Merck Millipore, Billerica, MA, USA) supplemented with protease and phosphatase inhibitor cocktails (Roche). Protein concentrations were determined with the BCA Protein Assay (Pierce Biotechnology, Rockford, IL, USA) according to the manufacturer’s instructions. Proteins were separated by SDS-polyacrylamide gel electrophoresis (PAGE) and then transferred to polyvinylidene difluoride (PVDF) membranes (Merck Millipore). After incubation with blocking solution (0.1 M phosphate buffer solution (PBS), 5% non-fat milk, 0.2% Tween-20), membranes were probed with primary antibodies (1:1000) at 4 °C overnight. The following primary antibodies were used: phospho-EGFR (3777; Cell Signaling Technology, Beverly, MA, USA), β-actin (4970; Cell Signaling Technology), GAPDH (5174; Cell Signaling Technology), phospho-ERK (4370; Cell Signaling Technology), ERK (4695; Cell Signaling Technology), phospho-STAT3 (9145; Cell Signaling Technology), phospho-STAT5 (4322; Cell Signaling Technology), α-Tubulin (3873; Cell Signaling Technology), phospho-AKT (4060; Cell Signaling Technology), AKT (2920; Cell Signaling Technology), and PCNA (SC-56; Santa Cruz Biotechnology, Santa Cruz, CA, USA). Membranes were then washed and incubated with secondary antibodies (1:10,000) (GE Healthcare, Waukesha, WI, USA). Then, the blots were developed with the ECL reagent (GE Healthcare Amersham, UK) and visualized by Azure c600 (Azure Biosystems, Dublin, CA, USA).

### 4.8. Chicken Chorioallantoic Membrane (CAM) Assay

Fertilized White Leghorn chicken eggs (50 ± 2 g) were incubated at 37.5 °C and 55% relative humidity. On day 3 of post-incubation, 4 mL albumin was withdrawn using a 21-gauge needle. On day 9, a 1 cm^2^ window was carefully created in the eggshell opposite the blunt edge and sealed with parafilm to prevent dehydration. The treatments, including the vehicle (ddH_2_O), TP2-5 (5 μg), or TP2-6 (5 μg), were separately air-dried on glass coverslips. bFGF (50 ng; Peprotech, Rocky Hill, NJ, USA) was used as a positive control. On day 10, the window was re-opened, and the coverslip was inverted over the CAM. The window was closed again, and the eggs were incubated for another 2 days. The window was opened on the 13th day, and vessels in the area under the coverslip were inspected and photographed under a microscope (Leica Microsystems, Wetzlar, Germany). According to the method used in a previous study [59], the number of vessel branch points was counted. The counts were performed double-blind and independently by two investigators.

### 4.9. Mouse Wound Healing Model

The animal study was approved by the Institutional Animal Care and Use Committee of Academia Sinica (Protocol # 21-06-1686, 06/22/2021). Thirty-two male BALB/c mice (8-week-old) were divided into four groups and individually anesthetized using an intraperitoneal injection of Zoletil 50 (50 mg/kg). The dorsal surface of each mouse was shaved. Then, a 5 mm punch biopsy tool was used to make identical full-thickness wounds on the dorsal skin of the mice. There were four treatment groups. Vehicle PBS (Veh), TP2-5 (2 μg/wound), TP2-6 (2 μg/wound), or EGF (2 μg/wound) [60] was applied four times every day. The open wound area was analyzed by tracing the wound margins in photographs taken on days 0, 2, 4, 6, 8, and 10. The wound areas were calculated (in mm^2^) using ImageJ software (*n* = 5). The skin around the wound was collected on day 4 post-surgery, and flash-frozen with liquid nitrogen (*n* = 3). The skin tissues were then stored at −80 °C for use in subsequent experiments.

### 4.10. Statistical Analysis

All values are displayed as mean ± SD. Multiple comparisons were performed with GraphPad Prism Software by one-way or two-way analysis of variance (ANOVA). Significant intergroup differences were subsequently tested by Bonferroni’s multiple comparison tests. The results were considered statistically significant for *p* values less than 0.05.

## 5. Conclusions

In this study, we found that TP2-5 and TP2-6 are bioactive compounds that enhance wound healing through the promotion of the proliferation and migration of keratinocytes and fibroblasts, the stimulation of collagen synthesis and KGF expression in fibroblasts, and the promotion of endothelial cell migration and angiogenesis. These results provide evidence that TP2-5 and TP2-6 may have utility as novel agents promoting wound healing.

## Data Availability

Data is contained within the article or Supplementary Materials.

## Supplementary Materials

The following supporting information can be downloaded at https://www.mdpi.com/article/10.3390/md20030205/s1, Figure S1: Inhibition of TP2-5- and TP2-6-induced epidermal growth factor receptor (EGFR) activation by small-molecule tyrosine kinase inhibitors. Serum-starved HaCaT cells were pretreated with the tyrosine kinase inhibitors, Gefitinib (10 µM) or PD158780 (10 µM), for 2 h and then incubated with 3.91 μg/mL of TP2-5 (A) or TP2-6 (B) for 24 h. Phosphorylated EGFR (p-EGFR) levels were detected by immunoblotting. α-tubulin and GAPDH were used as loading controls.
