# Supplementary material for: Investigations on the Wound Healing Potential of Tilapia Piscidin (TP)2-5 and TP2-6
Source: Mar Drugs. 2022 Mar 10;20(3):205. doi: 10.3390/md20030205 (PMC8955782; doi:10.3390/md20030205)
Supplement: Supplementary file 1 [file marinedrugs-20-00205-s001.zip › marinedrugs-1605360-supplementary.pdf]

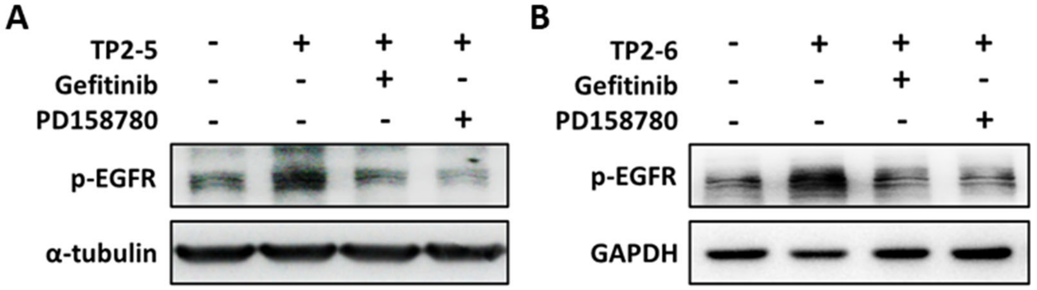

Figure S1. Inhibition of TP2-5- and TP2-6-induced epidermal growth factor receptor (EGFR) activation by small-molecule tyrosine kinase inhibitors. Serum-starved HaCaT cells were pretreated with tyrosine kinase inhibitors, Gefitinib (10 μM) or PD158780 (10 μM), for 2 h and then incubated with 3.91 μg/ml of TP2-5 (A) or TP2-6 (B) for 24 h. Phosphorylated EGFR (p-EGFR) levels were detected by immunoblotting. α-Tubulin and GAPDH were used as loading controls.
